# Preliminary data on the influence of rearing temperature on the growth and reproductive status of fathead minnows *Pimephales promelas*

**DOI:** 10.1111/j.1095-8649.2011.02993.x

**Published:** 2011-07

**Authors:** J V Brian, N Beresford, L Margiotta-Casaluci, J P Sumpter

**Affiliations:** Institute for the Environment, Brunel UniversityUxbridge, Middlesex UB8 3PH, U.K

**Keywords:** climate change, development, life history, reproduction, sex differentiation, sex ratio

## Abstract

An investigation into the influence of temperature on the growth and reproductive status of the fathead minnow *Pimephales promelas* revealed that, while there was no clear effect of treatment on sex differentiation, ovarian tissue from female fish reared under the highest temperature regime contained large amounts of undefined tissue containing no germ cells. Furthermore, both male and female fish exhibited differences in length mass, condition and somatic indices, and in the expression of secondary sexual characteristics. The patterns observed are discussed in the context of climate change.

## Introduction

Climate change has important implications for freshwater fishes, potentially exerting effects at all levels of biological organization, from the cellular, individual and population, through to the species, community and ecosystem levels (Graham & Harrod, 2009). Although it is difficult to predict its effects due to the complexities and uncertainties involved, it is fair to say that the primary effects on fishes will be mediated *via* changes in water temperature. Most fishes are ectothermic, which means that body temperature conforms to that of the surrounding environment. Water temperature therefore dictates the rate of all physiological processes, from molecular responses (*e.g.* the rate of vitellogenin gene expression; Brian *et al.*, 2008; Korner *et al.*, 2008), through to the ecological (*e.g.* the timing of reproduction; Gillet & Quétin, 2006).

Temperature also plays an important role in sex determination in some fish species (>60 species have been identified, from very divergent orders; Baroiller *et al.*, 2009). There is growing evidence, however, to suggest that, even for species in which sex is determined genetically (as opposed to environmentally), the processes involved are somewhat sensitive to temperature. For example, recent studies on the medaka *Oryzias latipes* (Temminck & Schlegel 1846) have revealed that individuals that are genetically female can be sex-reversed into the male phenotype by high temperature treatment during the period of sex differentiation (Sato *et al.*, 2005; Hattori *et al.*, 2009). This temperature-induced masculinization is thought to be mediated *via* the elevation of cortisol level, which suppresses germ cell proliferation and follicle-stimulating hormone receptor gene transcription (Hayashi *et al.*, 2010).

While the evolutionary explanation for this developmental plasticity remains elusive, the growing evidence of a continuum between genetic and temperature dependent sex determination begs the question as to whether the current rise in water temperatures may affect the reproductive development of wild fish populations (Johnson *et al.*, 2009). In addition to leading to male-biased sex ratios, it is possible that subtle, temperature-mediated effects on sex hormone levels could influence the processes involved in sex differentiation, leading to an increase in the incidence of intersex (*i.e.* the simultaneous presence of both male and female sex cells). This phenomenon, which is not normal for gonochoristic (single sex) fishes, has significant implications, having been associated with reduced reproductive capacity and population failure, in both the laboratory and the field (Lange *et al.*, 2001; Kidd *et al.*, 2007).

The aim of this study was to generate some preliminary data concerning the influence of rearing temperature on the reproductive development of a cyprinid fish species. The fathead minnow *Pimephales promelas* Rafinesque 1820 provides an ideal focus for this research, being widely used as an ecotoxicological model. The natural geographical range of this species extends throughout much of North America and, as such, it experiences considerable variations in water temperature, both on spatial and temporal scales (Page & Brooks, 1991). In the present study, larval *P. promelas* were reared, through to sexual maturity, under varying thermal conditions. Female fish were then examined for evidence of temperature-induced masculinization. In addition, a suite of data pertaining to the growth and reproductive status of both males and females was collected and interrogated for evidence of temperature-dependent effects.

## Materials and methods

Several batches of eggs were collected from an in-house stock of *P. promelas*. All the eggs were then placed into a single 30 l aquarium, which was well aerated and supplied with dechlorinated water at a rate of 10 l h^−1^. This was maintained at a temperature of 26° C using an aquarium heater (Visi-Therm 200 Watt Heater; http://www.aquariumsystems.eu). After 3–4 days, the eggs began to hatch and, 1 week later, when all the viable larvae had emerged, the fry were randomly allocated to one of eight experimental tanks (50 per tank), also maintained at 26° C. The temperatures in these tanks were then altered by either increasing or decreasing the heater settings at a rate of 1° C per day, such that there were four treatments of 20, 24, 28 and 32° C, with a duplicate tank in each. All other conditions remained identical.

Initially, the larval fish were fed exclusively on Liquifry (Interpet; http://www.interpet.co.uk), before graduating onto newly hatched brine shrimp *Artemia* sp. Once they were of a sufficient size, the fry were fed to satiation on a diet of frozen adult *Artemia* sp. and flaked fish food, three times each day. Temperature was measured daily to ensure that it stayed within 1° C of the target and, simultaneously, the dissolved oxygen levels were monitored using an Oxi 340i Digital Meter and CellOx 325 Probe (WTW; http://www.wtw.de). Water quality variables (*i.e.* pH, ammonia, nitrite and nitrate) were monitored throughout. These conditions were maintained for 6 months, at which point it was possible to discern the sex of the majority of fish by eye.

At the end of the rearing period, the fish were sacrificed with an overdose of anaesthetic (MS-222, Sigma-Aldrich; http://www.sigmaaldrich.com). Fork length (*L*_F_) and wet mass (*M*_W_) were recorded and sex assigned on the basis of an external examination. The condition factor (*K*) was determined from 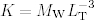. In females, the length of the ovipositor, which is a tubular structure that is used in egg deposition, was measured using digital callipers (Mahr 16ES, Mahr; http://www.mahr.de). The trunk was then placed in Bouin's fixative for subsequent histological examination of the ovaries, which were scored, based on the developmental stage of cells, in accordance with the criteria set out by the U.S. Environmental Protection Agency (USEPA, 2006). Male fish were analysed in terms of the number and prominence of nuptial tubercles (Smith, 1978), and the height of the mucous-secreting dorsal fatpad, which was scored according to the following criteria: 0, no visible fatpad; 1, small fatpad, raised <1 mm from body surface; 2, fatpad raised 2–5 mm; 3, fatpad raised >5 mm; 4, fatpad raised >5 mm, with folds (K. Thorpe, pers. comm.). As male tissues were not required for histological analysis, livers and gonads were then collected for the determination of hepato-somatic and gonado-somatic indices (*I*_H_ and *I*_G_).

While many of the data generated were purely qualitative, it was possible to analyse the biometric data using standard statistical techniques. Data sets that were normally distributed, with homogeneous variances, were analysed using parametric methods: each pair of duplicate tanks was compared using a *t*-test and, given that there was no difference between them, the data were pooled. The pooled data for each treatment were then subject to ANOVA and *post hoc* comparisons. Non-normal data sets were log_10_-transformed prior to analysis and, where normality could not be achieved, the same approach was employed using the equivalent non-parametric test (*i.e.* Mann–Whitney *U*-test followed by Kruskall–Wallis). The data from each of the duplicate tanks are presented (as opposed to the pooled data for each treatment) in order to show the degree of consistency between the patterns observed.

## Results

The results revealed that the survival rates at the end of the experiment were variable, ranging between 22 and 62%, with the lowest and highest survival occurring in the 24 and 20° C treatment groups, respectively. Sex was discernable in all but three cases (two from the highest and one from the lowest temperature treatments). Histological analysis revealed that the gonads of these fish were small and immature. As such, they were classified as juveniles and omitted from subsequent analyses. The sex ratios in each tank did not deviate significantly from parity, ranging between 35 and 60% male and no clear trends were observed in relation to temperature. In addition, there was no evidence of abnormal gonad development in females reared at elevated temperatures. Hence, the data do not support the initial hypothesis that the processes responsible for sex determination and sex differentiation in *P. promelas* are vulnerable to disruption due to differences in rearing temperature.

**Fig. 1:**
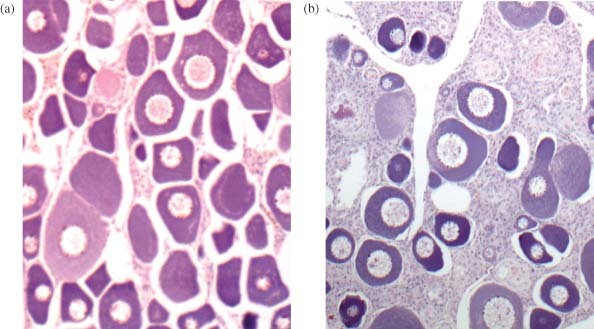
(a) Normal ovarian tissue sample from *Pimephales promelas*, with small quantities of interstitial tissue interspersed between oocytes and (b) an abnormal ovary from a female reared at an elevated temperature, which has a higher proportion of interstitial tissue and fewer germ cells (×100 magnification; H&E stain).

It may, however, be pertinent to note the abnormal presence of large amounts of interstitial tissue, containing no germ cells, in the ovaries of female fish reared in the higher temperature treatments (*n* = 5 at 32° C and *n* = 1 at 28° C). An example is shown in Fig. 1. In addition, the incidence of external developmental abnormalities (*e.g.* curvature of the spine, blindness in one or more eyes or abnormal fin development) was higher at elevated temperatures, such that the frequency of abnormalities was more than doubled in fish held at 32° C, relative to those held at lower temperatures. While these observations are purely qualitative, the incidence of the histological and morphological abnormalities outlined above suggests temperature-mediated effects on normal developmental processes and, thus, warrants further investigation.

**Fig. 2:**
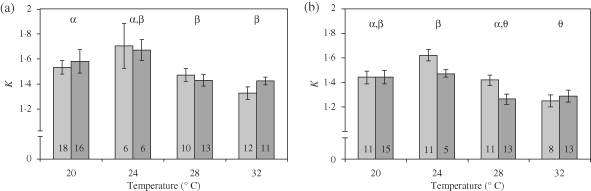
Mean ±s.e. body condition (*K*) of (a) male and (b) female *Pimephales promelas*, which were held within duplicate tanks (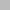, 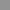), maintained under four different thermal regimes (20 to 32° C). Different symbols denote significant differences (*P* < 0·05) between treatment groups, based on ANOVA of pooled data. Sample sizes are given on the figure.

The biometric data also revealed differences between treatment groups. The mean *L*_F_ and *M*_W_ of fish in each treatment group are presented in Table I. There were significant differences in the *L*_F_ of females held at the lowest temperature and those in the two intermediate treatments (ANOVA, *F* = 4·92, d.f. = 86,3, *P* < 0·01). Female *M*_W_ also differed with treatment, being greater in fish held at 24° C, relative to those at both 20 and 32° C (ANOVA, *F* = 5·52, d.f. = 86,3, *P* < 0·01). Males exhibited a similar pattern, with fish reared at 28° C achieving greater mean *L*_T_ and *M*_W_ than those held at 20 or 32° C (ANOVA, *F* = 5·78, d.f. = 91,3, *P* < 0·01; ANOVA, *F* = 6·60, d.f. = 91,3, *P* < 0·001, respectively). Significant differences in *K* were also apparent (females: ANOVA, *F* = 11·29, d.f. = 86,3, *P* < 0·001; males: ANOVA, *F* = 5·04, d.f. = 86,3, *P* < 0·01). This was consistently highest and lowest at 24 and 32° C, respectively (Fig. 2).

**Table I:** Mean ± s.e. fork length (*L*_F_) and body mass (*M*_W_) of male and female *Pimephales promelas* within each treatment group

	*L*_F_ (mm)		*M*_W_ (g)	
		
Treatment temperature (° C)	Male	Female	Male	Female
20	49·2 ± 0·6^α^	41·1 ± 0·6^α^	1·89 ± 0·13^α^	1·03 ± 0·05^α^
24	50·0 ± 0·9^α,β^	44·7 ± 0·9^β^	2·12 ± 0·18^α,β^	1·46 ± 0·10^β^
28	54·1 ± 0·6^β^	44·1 ± 0·6^β^	2·31 ± 0·11^β^	1·18 ± 0·05^α,β^
32	49·6 ± 0·9^α^	42·4 ± 0·9^α,β^	1·68 ± 0·06^α^	1·02 ± 0·07^α^

Different symbols denote significant differences (*P* < 0·05) between treatment groups, based on ANOVA. The data from the duplicate tanks were pooled.

**Fig. 3:**
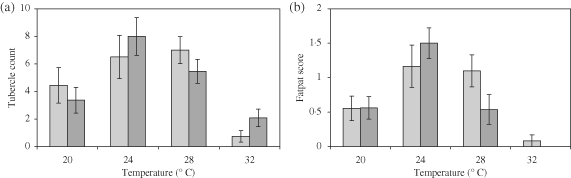
Mean ±s.e. (a) tubercle count and (b) fatpad score exhibited by male *Pimephales promelas*, which were held within duplicate tanks (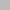, 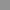), maintained under four different thermal regimes (20 to 32° C).

The analysis of secondary sexual characteristics also revealed differences between treatments. The relative ovipositor length of female fish maintained at 32° C was significantly lower than that of fish held at all other temperatures (ANOVA, *F* = 12·59, d.f. = 86,3, *P* < 0·001). Males differed in the appearance of their nuptial tubercles (number: Kruskall–Wallis, *H* = 26·03, d.f. = 3, *P* < 0·01; prominence: Kruskall–Wallis, *H* = 27·67, d.f. = 3, *P* < 0·01) and fatpad score (Kruskall–Wallis, *H* = 28·23, d.f. = 3, *P* < 0·01). The expression of these characteristics also appeared to be inhibited at 32° C (Fig. 3). In addition, it is interesting to note that the presence of a fin spot, which is another male secondary sexual characteristic, was reported at varying frequencies across the treatments (*χ*^2^ = 10·55, d.f. = 3, *P* < 0·05). This characteristic was always present in males held at 24° C and was least common in those at 32° C (present in only 15 of 23 males).

**Fig. 4:**
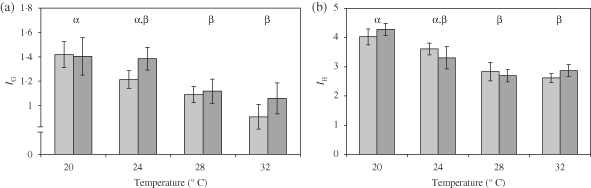
Mean ±s.e. (a) gonado-somatic index (*I*_G_) and (b) hepato-somatic index (*I*_H_) of male *Pimephales promelas*, which were held within the duplicate tanks (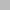, 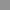) under each thermal regime (20 to 32° C). Different symbols denote significant differences (*P* < 0·05) between treatment groups, based on ANOVA of pooled data.

Differences in the *I*_H_ and *I*_G_ of male fish were also detected across treatments (ANOVA, *F* = 17·23, d.f. = 88,3, *P* < 0·001; ANOVA, *F* = 4·74, d.f. = 88,3, *P* < 0·01, respectively; Fig. 4). Unlike the other variables measured, both indices exhibited a linear relationship with temperature, declining as temperature increased. This means that, while these data support the assertion that elevated temperatures are sub-optimal in terms of growth and reproductive development, somatic indices were actually highest in males maintained at 20° C, rather than those held at an optimal temperature of 24° C.

## Discussion

The data generated during this study indicate that female *P. promelas* are not susceptible to temperature-induced masculinization under these experimental conditions. Variations in the thermal regime during development, however, did appear to influence growth and reproductive status in this species, such that the conditions in the 24° C treatment appeared optimal, with fish in these tanks exhibiting the lowest rate of deformity, having higher body condition and exhibiting well-developed sexual characteristics. Fish held at 28° C were similar in size to those held at 24° C, but were poorer in quality, having lower condition and somatic indices. In general, however, these fish were of good reproductive status, although fatpad size was slightly reduced relative to males held at 24° C. By contrast, the fish held at 20° C were smaller and the males less well developed, but were of the highest quality in terms of their somatic indices. The fish reared at 32° C were poor with respect to their growth, condition and reproductive status, as well as having the highest rate of deformities.

While there are clear differences between fish maintained under each thermal regime, it is important to recognize that rearing temperature was not the only variable that varied between treatment groups: differences in the rates of mortality during the early stages of development meant that there were also variations in density throughout the rearing period. Density-dependent effects, however, are likely to have been mitigated by the fact that (1) the tanks were not stocked excessively (the densities in all tanks were generally well below the maximum rate of one fish per litre recommended by the USEPA; Denny, 1988) and (2) competition for food was limited as fish were fed to excess. Furthermore, there was a remarkable consistency between the data sets from each pair of duplicate tanks, despite some differences in their densities. Hence, it is considered likely that the differences detected were, indeed, primarily a reflection of the thermal regime.

The patterns observed are consistent with evidence from bioenergetic studies, which show that the scope for growth in fishes is greatest in the middle of the temperature range and least at the extremes (Warren & Davis, 1967). In addition to having lower rates of growth, fishes reared under cooler conditions appeared to have delayed reproductive development, but invested more in their somatic development, whereas fishes reared under optimal or warm conditions did the opposite, allowing them to reproduce at a younger age. Presumably, these physiological trade-offs occur as plastic responses, which enable individuals to adopt alternative strategies, depending on the conditions encountered, and thereby maximize their fitness under different thermal regimes. It is interesting to note that the compensatory mechanisms appear to break down at higher temperatures, with an apparent threshold of between 28 and 32° C for *P. promelas*.

The patterns observed in this laboratory-based study are consistent with the findings of a meta-analysis of the life-history traits of 44 species of European freshwater fishes, which revealed that, while species at lower latitudes are often smaller, they grow faster and mature earlier than those at higher latitudes. They also have shorter life spans and allocate less of their energy to reproduction (Blanck & Lamouroux, 2007). Latitudinal differences have been reported within species. For example, there is evidence that Arctic charr *Salvelinus alpinus* (L. 1758) exhibit lower longevity, lower age at maturity, lower maximum size and increased growth rate with decreasing latitude, presumably in response to variations in water temperature (Jeppesen *et al.*, 2010).

In addition, the present data are consistent with the expectation that, while smaller adults emerge if growth is limited by food availability, most animals grow more slowly in cold conditions but reach a larger size than at high temperatures. Potential explanations for this apparent paradox are outlined by Atkinson & Sibly (1997): it may be that the response of adult size to temperature is adaptive, but is constrained by a trade-off that can be understood in terms of von Bertalanffy's classic theory of growth or that the response may be the unavoidable consequence of a fundamental relationship between cell size and temperature. In any case, it would appear that, in addition to latitudinal differences in the size structure of fish populations, which are indicative of long-term evolution and adaptation to the climatic conditions, changes in size and age structure can also occur over relatively short time scales.

In this respect, Jeppesen *et al.* (2010) analysed monitoring data from 200 Danish lakes, collected between 1989 and 2006, which revealed an increase in the proportion of small perch *Perca fluviatilis* L. 1758 and bream *Abramis brama* (L. 1758) with increasing summer temperature. This indicates that these species can respond rapidly to changes in climate, possibly *via* the same mechanisms as those responsible for the size differences reported here. In contrast, size structure in roach *Rutilus rutilus* (L. 1758) did not vary over the same period, suggesting that this species may be less able to respond to climate change. In addition to comparing how different species respond to increasing temperatures, it may be pertinent to consider the extent to which temperature influences the life history of populations within the same species, as their plasticity in this respect may vary throughout their geographical range.

With regard to the present study, which provides only a snapshot of the effects of temperature at one particular time point, it would be useful to repeat the experiment to assess the influence of temperature on the timing of developmental events. Measuring the time course of developmental changes would reveal whether the observed effects on reproductive status are due to general effects on rates of growth, or are unique to reproductive endpoints, or whether differences in temperature causes heterochronies in the various traits assessed. The experiment could also be extended to determine whether the fish in each treatment ultimately varied in terms of additional life-history characteristics, such as adult size, fecundity and longevity. Future studies should take care to exclude density as a confounding factor and should minimize mortality during early development, thereby ruling out the possibility of differences arising due to selective mortality.

An improved understanding of these issues would help to reveal whether temperature-mediated effects on growth and reproductive development are likely to affect the lifetime reproductive potential of fishes: while faster growth and earlier maturation may have a positive effect on fitness and fecundity in warmer waters, these benefits may be negated by the smaller body size attained, combined with reduced longevity (Jeppesen *et al.*, 2010). As such, the overall effect of increasing water temperature on recruitment may be negligible. The data presented here, however, indicates that there is a critical thermal threshold, above which growth and reproductive development are impaired, with likely consequences for population sustainability.

It is therefore concluded that, while *P. promelas* appears to be relatively thermo-tolerant and is able to survive and reproduce across a wide range of temperatures, variations in thermal conditions during development can affect a wide range of life-history characteristics. At high temperatures, the effects were profound, with the fish exhibiting poorer condition and reproductive status, as well as having an increased rate of histological and developmental abnormalities, but even at lower temperatures, there was evidence of subtle effects on rates of growth and reproductive development, which may potentially affect fitness and fecundity. These subtle, temperature-dependent effects on population-level variables may be of ecological significance, particularly in habitats that are already under pressure from pollution and overexploitation. For example, Jeppesen *et al.* (2010) predicted that climate-mediated effects on lake fish community structure, occurring, in part, *via* changes in life history, may increase the risk of eutrophication. Thus, further research is required to improve the understanding of how climate change influences life-history traits and to elucidate the consequences at the population and ecosystem level.
